# Urinary iodine-to-creatinine ratio and outcomes after radioactive iodine ablation in thyroid cancer

**DOI:** 10.1210/jendso/bvag125

**Published:** 2026-06-03

**Authors:** Maria Vamvini, Ashna Grover, Josephine H Li, Zhiheng H He, James V Hennessey

**Affiliations:** Division of Endocrinology, Diabetes, and Metabolism, Beth Israel Deaconess Medical Center, Harvard Medical School, Boston, MA 02215, USA; Joslin Diabetes Center, Harvard Medical School, Boston, MA 02215, USA; Department of Medicine, Mount Auburn Hospital, Harvard Medical School, Cambridge, MA 02138, USA; Diabetes Unit, Endocrine Division, Massachusetts General Hospital, Boston, MA 02114, USA; Department of Medicine, Harvard Medical School, Boston, MA 02115, USA; Division of Endocrinology, Diabetes, & Nutrition, Boston Medical Center-Brighton, Brighton, MA 02135, USA; Division of Endocrinology, Diabetes, and Metabolism, Beth Israel Deaconess Medical Center, Harvard Medical School, Boston, MA 02215, USA

**Keywords:** urinary iodine, thyroid cancer, radioactive iodine ablation

## Abstract

**Context:**

Radioactive iodine ablation (RAIA) in differentiated thyroid cancer (DTC) requires elevated thyroid-stimulating hormone (TSH) levels, achieved through thyroid hormone withdrawal (THW) or recombinant human TSH (rhTSH). A low-iodine diet (LID) is recommended to enhance RAIA efficacy, but its impact on long-term outcomes remains uncertain.

**Objective:**

To determine whether pre-ablation urinary iodine concentrations predict disease outcomes after RAIA.

**Methods:**

This retrospective study evaluated DTC patients treated with post-surgical RAIA at BIDMC from 2007 to 2020. Of 463 patients reviewed, 304 met inclusion criteria (THW: 189; rhTSH: 115), all completing a 2-week LID; urinary iodine-to-creatinine ratio (UICR) was measured as iodine status marker. Disease recurrence at 12 months, defined by elevated thyroglobulin (Tg), abnormal neck ultrasound, or a positive whole-body scan, was the primary outcome, with associations between UICR and outcomes evaluated using univariable and multivariable logistic regression.

**Results:**

Positive pre-treatment Tg (odds ratio [OR] 6.13, 95% CI 1.90-19.83; *P* = .002) and AJCC stage IV disease (OR 8.45, 95% CI 1.39-51.36; *P* = .02) were independently associated with recurrence. Older age was associated with lower recurrence risk only in unadjusted models. UICR showed no association with outcomes. Although mean UICR was higher in the rhTSH group (*P* < .0001), recurrence rates were similar between THW and rhTSH (*P* = .3).

**Conclusion:**

Higher UICR in rhTSH-prepared patients did not translate into worse outcomes. Pre-treatment Tg and stage IV disease were the strongest predictors of recurrence, whereas iodine status was not. These findings suggest that strict iodine depletion at the level achieved here, reflected by UICR, may not determine RAIA efficacy in DTC.

Radioactive iodine ablation (RAIA) is often administered after thyroidectomy in patients with differentiated thyroid cancer (DTC) to eliminate residual thyroid tissue and microscopic metastases using iodine-131 [1]. RAIA has been associated with improved disease-free survival, reduced recurrence, and lower mortality in patients at moderate to high risk of recurrence [2]. Adequate pre-ablation stimulation of thyroid-stimulating hormone (TSH) is essential to enhance I-131 uptake, achieved either through thyroid hormone withdrawal (THW) or administration of recombinant human TSH (rhTSH) [3]. While traditional protocols recommend achieving TSH levels above 25-30 mIU/L, recent evidence suggests that higher levels do not necessarily improve ablation success, and both THW and rhTSH preparations yield comparable oncologic outcomes and survival rates [4, 5].

A low-iodine diet (LID), typically restricting intake to less than 50 µg/day, is routinely recommended before RAIA to deplete body iodine stores and increase the effectiveness of I-131 via upregulation of sodium–iodide symporters (NIS) [6, 7]. However, LID can be difficult for patients to follow, often leading to poor adherence. The urinary iodine-to-creatinine ratio (UICR) is a widely accepted, noninvasive biomarker for assessing iodine status and LID compliance. It also helps guide the timing of RAIA, particularly after exposure to iodine-containing agents such as contrast media or amiodarone. Elevated urinary iodine levels (eg, >250 µg/gCr) at the time of ablation have been associated with reduced treatment success [8].

Despite its widespread use, the impact of LID on RAIA outcomes remains uncertain due to inconsistent urinary iodine monitoring and limited data on patients prepared with rhTSH rather than THW [9]. Moreover, whether RAIA should be postponed in patients with elevated UICR levels remains an unresolved clinical question. This study aims to evaluate the relationship between iodine depletion status, as measured by UICR, and RAIA outcomes, and to determine whether this association varies by method of TSH stimulation.

## Materials-methods

### Study design

This was a retrospective study conducted at Beth Israel Deaconess Medical Center (BIDMC), evaluating patients between 2007 and 2020. The study protocol was approved by the Institutional Review Boards of both BIDMC and Dana-Farber Cancer Institute.

### Participants

**Figure 1:**
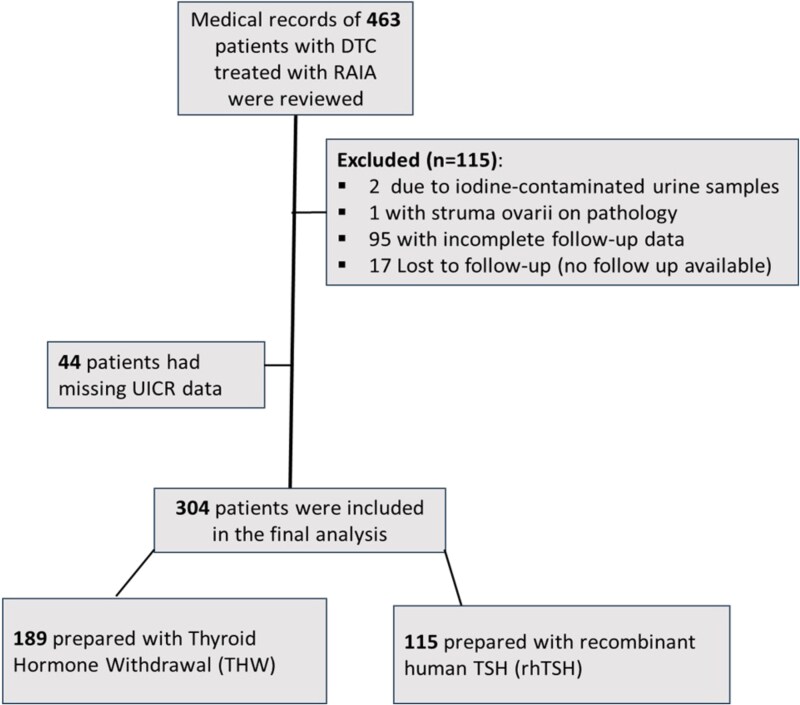
Study flow diagram showing patient inclusion and exclusion.

We retrospectively reviewed charts of 463 patients with DTC who underwent total thyroidectomy and initial RAIA at the BIDMC from 2007 to 2020. All patients regardless of age, sex, self-reported race, educational background, and socioeconomic status were included. Patients with undifferentiated anaplastic and medullary thyroid cancers, thyroid lymphoma, and those who had initial ablation before July 1, 2007 were excluded. After excluding 159 patients due to loss to follow-up, incomplete data, or missing pre-ablation urinary iodine measurements, 304 patients were included in the final analysis (THW: *n* = 189; rhTSH: *n* = 115) (Fig. 1). Tumor classification was determined according to the TNM system, and overall disease stage was assigned based on the American Joint Committee on Cancer (AJCC) 8th edition criteria [10].

### Low-iodine diet and RAIA preparation protocol

All patients were instructed to follow a 2-week LID prior to RAIA. The diet aimed to restrict daily iodine intake to less than 50 µg to enhance uptake of radioactive iodine by remnant thyroid tissue. Instructions were provided during clinic visits by a physician and in written form by an administrative assistant as a standardized educational leaflet detailing food restrictions. Patients were advised to avoid iodized salt, dairy products, seafood, processed foods with iodine additives, and certain food dyes. The LID was self-managed by the patient. To assess iodine depletion and LID compliance, the UICR was measured while on the LID and on the Friday prior to RAIA. The UICR served as a surrogate marker for total body iodine status. The LID was continued until the Saturday morning following the 131-I ablation which typically occurred on a Wednesday.

Patients were prepared for RAIA using one of 2 standardized methods: thyroid hormone withdrawal (THW) or recombinant human TSH (rhTSH) stimulation. The preparation method was selected by the treating endocrinologist based on clinical factors, patient preference, and rhTSH availability. For the THW protocol, levothyroxine therapy was discontinued 6 weeks before RAIA. Patients received triiodothyronine substitution for the first 4 weeks, followed by complete thyroid hormone withdrawal for the subsequent 2 weeks to induce endogenous TSH elevation, targeting a pre-ablation TSH concentration exceeding 25-30 mIU/L. The goal of achieving a TSH >25 mIU/mL pertained only to patients prepared by THW. For the rhTSH protocol, patients received 2 consecutive daily intramuscular injections of 0.9 mg rhTSH (thyrotropin alfa; Thyrogen, Genzyme Corp., Cambridge, MA) in accordance with standard institutional procedures, Injections typically occurred on a Monday and Tuesday and the RAIA was administered 24 hours after the second injection. In both groups, serum TSH, and UICR were measured immediately before ablation to confirm adequate iodine depletion. Patients undergoing THW routinely had stimulated thyroglobulin (Tg) also assessed. Nonstimulated Tg (while on Levothyroxine) was not always obtained prior to rhTSH-stimulated planned ablations.

### Outcome definition

The primary outcome was disease recurrence/persistence at 12 months following initial RAIA. Disease recurrence/persistence was defined by the presence of any of the following: (a) positive serum Tg level, either basal or TSH-stimulated, above the assay-specific reference threshold, (b) structural evidence of disease on neck ultrasound, or (c) uptake consistent with residual or recurrent disease on whole-body radioiodine scan. Patients who did not meet any of these criteria were classified as disease-free.

### Statistical analysis

Continuous variables are presented as mean ± SD (normal distribution) or as median with interquartile range (skewed distribution), while categorical variables are presented as percentages. Statistical comparisons were performed using the Pearson's χ^2^ test, unpaired t test and Mann–Whitney *U* test, where appropriate. Continuous variables were assessed for normality. UICR values were log-transformed due to right-skewed distribution. Pre-treatment TSH levels and administered I-131 doses were categorized into 3 groups (<25 mIU/L, 25-100 mIU/L, and >100 mIU/L; and ≤50 mCi, 51-100 mCi, and >100 mCi, respectively). Pre-treatment Tg and thyroglobulin antibody (TgAb) levels were analyzed as binary variables (positive vs negative). Because the study spanned several years, Tg levels were measured using different assays with varying sensitivity thresholds; therefore, Tg was considered positive when it was detectable and exceeded the assay-specific lower limit.

Univariable analyses were first performed to compare patients according to disease status during follow-up (recurrence/persistence vs disease-free). Similar unadjusted comparisons were then conducted based on the preparation method (THW vs rhTSH). Subsequently, comparisons by disease status were repeated within each preparation method. Two multivariable logistic regression models were constructed with disease status (recurrence/persistence) as the outcome. Model 1 included age, UICR (log-transformed), preparation method, stage, and tumor characteristics. Model 2 included the same covariates as Model 1, with the addition of pre-treatment Tg status. Sex was evaluated for inclusion but was excluded due to lack of association with the outcome and constraints on model degrees of freedom. To assess potential effect modification by preparation method, interaction terms between preparation type and other model covariates were jointly tested. Because some interaction terms were dropped due to perfect prediction, penalized (Firth's) logistic regression was employed for the interaction analysis. Statistical significance was defined as a 2-sided *P*-value <.05. All analyses were conducted using Stata Statistical Software, Release 13 (StataCorp LP, College Station, TX).

## Results

### Baseline characteristics

Baseline characteristics of the study cohort are summarized in Table 1. The mean age was 49.5 years (SD 15.2), and 27% of patients were female. Most patients (67%) were White, followed by Asian (18%) and Black (6%). Papillary thyroid carcinoma was the predominant histologic subtype (93%), with follicular (4%) and Hürthle cell (3%) variants comprising the remainder. According to the AJCC 8th edition staging system, 58% of patients presented with Stage I disease, 15% with Stage II, 24% with Stage III, and 3% with Stage IV. Based on the TNM classification, 42% of tumors were classified as T1, 28% as T2, 29% as T3, and 1% as T4. Lymph node metastases (N1) were present in 27% of patients, and distant metastases (M1) were identified in 6%. A total of 189 patients (62%) were prepared with THW and 115 (38%) with rhTSH. Pre-treatment TSH levels were <25 mIU/L in 37%, 25-100 mIU/L in 38%, and >100 mIU/L in 25% of patients. Pre-treatment Tg was positive in 61%, and TgAbs were positive in 12%. Most patients (88%) received an I-131 dose of ≤100 mCi. The median UICR was 41.3 µg/g (IQR 22.4-73.6), with a mean log-transformed UICR of 3.75 (SD 0.93). The median follow-up duration was 12 months (IQR 10-14), during which 40 patients (13%) demonstrated disease recurrence or persistence.

**Table 1:** Baseline characteristics of the cohort

Characteristic	Total (*n* = 304)
**Demographics**	
Female, *n* (%)	83 (27)
Age, mean (SD), years	49.5 (15.2)
Race, *n* (%)	
White	203 (67)
Asian	54 (18)
Black	18 (6)
Unknown	24 (8)
Other	5 (1)
**Tumor characteristics**	
Type, *n* (%)	
Papillary	284 (93)
Follicular	11 (4)
Hurthle	9 (3)
Stage, (AJCC), *n* (%)*^a^*	
I	177 (58)
II	45 (15)
III	73 (24)
IV	8 (3)
Tumor (T), stage, *n* (%)*^b^*	
T1	127 (42)
T2	86 (28)
T3	87 (29)
T4	2 (1)
Node (N), stage, *n* (%)*^b^*	
N0	98 (33)
N1	82 (27)
Nx	123 (40)
Metastasis (M), stage, *n* (%)*^b^*	
M0	283 (94)
M1	19 (6)
Size in cm, median (IQR)	2 (1.2-3)
**Treatment preparation and biochemical variables**	
Preparation method, *n* (%)	
THW	189 (62)
rhTSH	115 (38)
Pre-treatment TSH level, *n* (%)	
<25 mIU/L	112 (37)
25-100 mIU/L	115 (38)
>100 mIU/L	77 (25)
Pre-treatment Tg status, *n* (%)*^c^*	
Negative	110 (39)
Positive	170 (61)
Pre-treatment Tgab status, *n* (%)*^d^*	
Positive	35 (12)
Negative	259 (88)
I 131 dose, *n* (%)	
≤50 mCi	146 (48)
51-100 mCi	122 (40)
>100 mCi	36 (12)
Urine iodine, median (IQR), µg/L	39 (19-79.5)
UICR, median (IQR)	41.28 (22.44-73.57)
UICR, log-transformed, mean (SD)	3.75 (0.93)
UICR quartiles, median (IQR), µg/gCr	
Q1	14.84 (10.12 -18.70)
Q2	32.27 (27.73-37.05)
Q3	54.64 (47.82-64.34)
Q4	119.01 (90.68-176.15)
Time to follow-up, days median (IQR)	358 (293-437)
Disease recurrence/persistence, *n* (%)	40 (13)

^*a*^1 missing observation.

^*b*^2 missing observations.

^*c*^24 missing observations.

^*d*^10 missing observations.

### Association of UICR with disease status

UICR did not differ significantly between patients with disease recurrence/persistence and those who were disease-free (log-transformed mean ± SD: 3.64 ± 0.99 vs 3.76 ± 0.92, *P* = .44). Median urinary iodine concentrations were likewise similar between groups (38.5 [19-76.5] µg/L vs 44.5 [21.5-94] µg/L, *P* = .61). Similarly, when UICR was analyzed by quartiles, there was no association between UICR category and disease recurrence/persistence (*P* > .05) (data not shown). As shown in Table 2, patients who experienced disease recurrence/persistence were younger (45.0 ± 18.1 vs 50.3 ± 14.7 years, *P* = .04) and more likely to present with advanced AJCC stage (Stage III-IV: 35% vs 26%, *P* = .01) and lymph-node metastases (N1: 61.5% vs 22%, *P* < .0001). Positive pre-treatment Tg status was also more frequent among patients with recurrence/persistence (89% vs 57%, *P* < .0001), as were higher administered I-131 doses (*P* < .0001). There were no significant differences between groups by sex, preparation method (THW vs rhTSH), or pre-treatment TSH levels.

**Table 2:** Univariable comparison of baseline characteristics between patients with disease recurrence/persistence and those who were disease-free at 12 months

Characteristic	Recurrence/persistence (*n* = 40)	Disease-free (*n* = 264)	*P* value
**Demographics**			
Female, *n* (%)	26 (65)	195 (75)	.26
Age, mean (SD), years	45.0 (18.1)	50.3 (14.7)	.04
Race, *n* (%)			
White	28 (70)	175 (66)	.85
Asian	7 (17.50)	47 (18)	
Black	1 (2.5)	17 (6.5)	
Unknown	3 (7.5)	21 (8)	
Other	1 (2.5)	4 (1.5)	
**Tumor characteristics**			
Type, *n* (%)			.36
Papillary	40 (100)	244 (92.5)	
Follicular	0	11 (4)	
Hurthle	0	9 (3.5)	
Stage, (AJCC), *n* (%)			.01
I	24(60)	153 (58)	
II	2 (5)	43 (16)	
III	10 (25)	63 (24)	
IV	4 (10)	4 (2)	
Tumor (T), stage, *n* (%)			.17
T1	14 (36)	113 (43)	
T2	9 (23)	77 (29)	
T3	15 (38.5)	72 (27)	
T4	1 (2.5)	1 (1)	
Node (N), stage, *n* (%)			<.0001
N0	8 (20.5)	90 (34)	
N1	24 (61.5)	58 (22)	
Nx	7 (18)	115 (44)	
Size in cm, median (IQR)	1.7 (1.2-2.6)	2 (1.2-3.15)	.42
**Treatment preparation and biochemical variables**			
Preparation method, *n* (%)			.3
THW	28 (70)	161 (61)	
rhTSH	12 (30)	103 (39)	
Pre-treatment TSH level, *n* (%)			.6
<25 mIU/L	12 (30)	100 (38)	
25-100 mIU/L	16 (40)	99 (37)	
>100 mIU/L	12 (30)	65 (25)	
Pre-treatment Tg status, *n* (%)			<.0001
Negative	4 (11)	106 (43)	
Positive	32 (89)	138 (57)	
Pre-treatment Tgab status, *n* (%)			.11
Positive	8 (20.5)	27 (11)	
Negative	31 (79.5)	228 (89)	
I 131 dose, *n* (%)			<.0001
≤50 mCi	8 (20)	138(52)	
51-100 mCi	17 (42.5)	105 (40)	
>100 mCi	15 (37.5)	21 (8)	
UICR, log-transformed, mean (SD)	3.64 (0.99)	3.76 (0.92)	.44
Urine iodine, median (IQR)	38.5 (19-76.5)	44.5 (21.5-94)	.61

### Differences in UICR and biochemical characteristics by preparation method

A comparison of UICR and related clinical variables between patients prepared with THW and rhTSH is shown in Table 3. Urinary iodine concentrations were markedly lower in the THW group (median [IQR] 26 [14-64] µg/g vs 63 [34-111] µg/g, *P* < .0001), consistent with greater iodine depletion achieved through thyroid hormone withdrawal. Correspondingly, the mean log-transformed UICR was significantly lower in THW-prepared patients (3.4 ± 0.9 vs 4.3 ± 0.6, *P* < .0001). In the THW group, the majority of patients successfully achieved a TSH >25 mIU/mL, confirming that the preparation protocol was appropriately followed. Only 7 THW patients had a TSH <25 mIU/mL. In the rhTSH-prepared group, TSH levels were obtained in all patients to assess whether they were meeting TSH goals per ATA guidelines while on LT4 therapy. As expected, the majority had TSH levels <25 mIU/mL. As expected, pre-treatment TSH levels were substantially higher among THW-prepared patients (*P* < .0001), reflecting endogenous stimulation, while pre-treatment Tg positivity was also more common (77% vs 34%, *P* < .0001). Although racial distribution differed slightly between groups (*P* = .01), other baseline and tumor characteristics, including age, sex, histology, AJCC stage, and TNM classification, were comparable. I-131 doses were generally higher in the THW group (*P* < .0001), yet rates of disease recurrence/persistence at 12 months did not differ significantly between preparation methods (*P* = .30).

**Table 3:** Comparison of characteristics and biochemical variables between patients prepared with THW and rhTSH

Characteristic	THW (*n* = 189)	rTSH (*n* = 115)	*P* value
**Demographics**			
Female, *n* (%)	135 (71)	86 (75)	.6
Age, mean (SD), years	48.6 (14.8)	51.2 (15.8)	.15
Race, *n* (%)			.01
White	123 (65)	80 (70)	
Asian	34 (18)	20 (17)	
Black	15 (8)	3 (3)	
Unknown	17 (9)	7 (6)	
Other	0 (0)	5 (4)	
**Tumor characteristics**			
Type, *n* (%)			.18
Papillary	180 (95)	104 (90)	
Follicular	4 (2)	7 (6)	
Hurthle	5 (3)	4 (3)	
Stage, (AJCC), *n* (%)			.79
I	108 (57)	69 (61)	
II	27 (14)	18 (16)	
III	48 (25)	25 (22)	
IV	6 (3)	2 (2)	
Tumor (T), stage, *n* (%)			.36
T1	73 (39)	54 (47)	
T2	54 (29)	32 (28)	
T3	59 (31)	28 (25)	
T4	2 (0)	0 (0)	
Node (N), stage, *n* (%)			.69
N0	61 (32)	37 (32)	
N1	50 (27)	32 (28)	
Nx	77(41)	45 (40)	
Size in cm, median (IQR)			
**Treatment preparation and biochemical variables**			
Disease recurrence, *n* (%)	28 (15)	12 (10)	.3
Pre-treatment TSH level, *n* (%)			<.0001
<25 mIU/L	7 (4)	105 (91)	
25-100 mIU/L	110 (58)	5 (4)	
>100 mIU/L	72 (38)	5 (4)	
Pre-treatment Tg status, *n* (%)			<.0001
Negative	41 (23)	69 (66)	
Positive	135 (77)	35 (34)	
Pre-treatment Tgab status, *n* (%)			1
Positive	163 (88)	96 (88)	
Negative	22 (12)	13 (12)	
I 131 dose, *n* (%)			<.0001
≤50 mCi	83 (44)	63 (55)	
51-100 mCi	73 (39)	49 (43)	
>100 mCi	33 (17)	3 (2)	
UICR, log-transformed, mean (SD)	3.4 (0.9)	4.3 (0.6)	<.0001
Urine iodine, median (IQR)	26 (14-64)	63 (34-111)	<.0001

### Relationship between UICR and disease recurrence/persistence within preparation subgroups

Stratified analyses by preparation method are presented in Table 4. Among patients prepared with THW, the median UICR and urinary iodine concentrations did not differ significantly between those with disease recurrence/persistence and those who were disease-free (log-transformed UICR: 3.34 ± 1.03 vs 3.42 ± 0.90, *P* = .69; urinary iodine: 28.5 [10-79] vs 26 [14-60] µg/L, *P* = .77). Patients with recurrence/persistence tended to have more advanced disease, with a higher proportion of N1 stage (63% vs 21%, *P* < .0001) and slightly more T3/T4 tumors (*P* = .06). Pre-treatment Tg positivity was common in both groups (89% vs 75%), though not significantly different (*P* = .14). Higher administered I-131 doses were observed among patients with recurrence/persistence (*P* < .0001), while age, sex, and pre-treatment TSH levels were similar between groups (Table 4).

**Table 4:** Baseline and biochemical characteristics by disease status in the 2 different RAIA prep groups

	THW group			rhTSH group		
Characteristic	Recurrence/persistence (*n* = 28)	Disease-free (*n* = 161)	*P*	Recurrence/persistence (*n* = 12)	Disease-free (*n* = 103)	*P*
**Demographics**						
Female, *n* (%)	19 (68)	116 (72)	.66	7 (58)	79 (77)	
Age, mean (SD), years	46.5 (16.6)	48.9 (14.5)	.43	41.4 (21.4)	52.3 (14.7)	.02
Race, *n* (%)			.06			.01
White	21 (75)	102 (63)		7 (58)	73 (71)	
Asian	7 (25)	27 (17)		0 (0)	20 (19)	
Black	0 (0)	15 (9)		1 (8)	2 (2)	
Unknown	0 (0)	17 (11)		3 (25)	4 (4)	
Other	0 (0)	0 (0)		1 (8)	4 (4)	
**Tumor characteristics**						
Type, *n* (%)			1			1
Papillary	28 (100)	152 (94.5)		12 (100)	92 (89)	
Follicular	0 (0)	4 (2.5)		0 (0)	7 (7)	
Hurthle	0 (0)	5 (3)		0 (0)	4 (4)	
Stage, (AJCC), *n* (%)			.09			.004
I	15 (54)	93 (58)		9 (75)	60 (59)	
II	1 (4)	26 (16)		1 (8)	17 (17)	
III	10 (36)	38 (24)		0 (0)	25 (25)	
IV	2 (7)	4 (2)		2 (17)	0 (0)	
Tumor (T), stage, *n* (%)			.06			.58
T1	9 (33)	64 (40)		5 (42)	49 (48)	
T2	4 (15)	50 (31)		5 (42)	27 (26)	
T3	13 (48)	46 (28)		2 (16)	26 (26)	
T4	1 (4)	1 (1)		0 (0)	0 (0)	
Node (N), stage, *n* (%)			<.0001			.11
N0	5 (18.5)	56 (35)		34 (33)	3 (25)	
N1	17 (63)	33 (21)		25 (25)	7 (58)	
Nx	5 (18.5)	72 (45)		43 (42)	2 (17)	
**Treatment preparation and biochemical variables**						
Pre-treatment TSH level, *n* (%)			.87			.68
<25 mIU/L	1 (4)	6 (4)		11 (92)	94 (91)	
25-100 mIU/L	15 (54)	95 (59)		1 (8)	4 (4)	
>100 mIU/L	12 (43)	60 (37)		0 (0)	5 (5)	
Pre-treatment Tg status, *n* (%)			.14			<.0001
Negative	3 (11.5)	38 (25)		1 (10)	68 (72)	
Positive	23 (88.5)	112 (75)		9 (90)	26 (28)	
Pre-treatment Tgab status, *n* (%)			.11			.62
Positive	6 (21)	16 (10)		2 (18)	11 (11)	
Negative	22 (79)	141 (90)		9 (82)	87 (89)	
I 131 dose, *n* (%)			<.0001			.005
≤50 mCi	5 (18)	78 (48.5)		3 (25)	60 (58)	
51-100 mCi	10 (36)	63 (39)		7 (58)	42 (41)	
>100 mCi	13 (46)	20 (12.5)		2 (17)	1 (1)	
UICR, log-transformed, mean (SD)	3.34 (1.03)	3.42 (0.90)	.69	4.32 (0.45)	4.30 (0.67)	.88
Urine iodine, median (IQR)	28.5 (10-79)	26 (14-60)	.77	72 (41-291.5)	63 (34-110)	.29

In the rhTSH-prepared subgroup, neither UICR nor urinary iodine levels were associated with disease recurrence/persistence (log-transformed UICR: 4.32 ± 0.45 vs 4.30 ± 0.67, *P* = .88; urinary iodine: 72 [41-291] vs 63 [34-110] µg/L, *P* = .29). Patients with recurrence/persistence were younger (41.4 ± 21.4 vs 52.3 ± 14.7 years, *P* = .01), more often female (*P* = .02), and presented with more advanced AJCC stage (*P* = .004). Pre-treatment Tg positivity (90% vs 28%, *P* < .0001) and higher I-131 doses (*P* = .005) were also associated with recurrence/persistence in this group (Table 4).

Overall, UICR did not differ significantly between patients with and without disease recurrence/persistence in either preparation method.

### Multivariable analysis of predictors of disease recurrence/persistence

Two multivariable logistic regression models were constructed to identify independent predictors of disease recurrence/persistence (Table 5). In Model 1, which included age, log-transformed UICR, preparation method (rhTSH vs THW), AJCC stage, and tumor (T) stage, older age remained independently associated with a lower likelihood of disease recurrence/persistence (odds ratio [OR] 0.97, 95% CI 0.94-0.99, *P* = .02). UICR showed no association with disease recurrence/persistence (OR 0.99, 95% CI 0.66-1.50, *P* = .99), and the preparation method was not a significant predictor (OR 0.85, 95% CI 0.36-1.97, *P* = .70). Compared with AJCC Stage I, patients with Stage IV disease had a substantially greater risk of recurrence/persistence (OR 13.89, 95% CI 2.44-78.42, *P* = .03). Tumor T stage was not independently associated with outcome after adjustment for other variables. In Model 2, pre-treatment Tg status was added to the model. Positive pre-treatment Tg emerged as a strong independent predictor of disease recurrence/persistence (OR 6.13, 95% CI 1.90-19.83, *P* = .002). The association between Stage IV disease and recurrence/persistence remained significant (OR 8.45, 95% CI 1.39-51.36, *P* = .02), whereas the effects of age, UICR, and preparation method were not statistically significant. Overall, across both models, UICR was not independently associated with disease recurrence/persistence.

**Table 5:** Multivariable logistic regression analysis of factors associated with disease recurrence/persistence

	Model 1			Model 2		
Variable	Odds ratio	95% CI	*P* value	Odds ratio	95% CI	*P* value
Age, years	0.97	0.94-0.99	.02	0.97	0.94-1.00	.08
UICR, log-transformed	0.99	0.66-1.5	.99	1.04	0.66-1.62	.87
Preparation method (rhTSH)	0.85	0.36-1.97	.7	1.4	0.52-3.77	.5
Stage, (AJCC)						
I	ref			ref		
II	0.46	0.08-2.6	.38	0.59	0.95-3.68	.57
III	1.25	0.38-4.09	.71	1.21	0.32-4.55	.78
IV	13.89	2.44-78.42	.03	8.45	1.39-51.36	.02
Tumor (T), stage						
T1	ref			ref		
T2	1.09	0.39-3.08	.87	0.73	0.23-2.35	.61
T3	1.76	0.67-4.63	.26	1.48	0.52-.4.25	.47
T4	2.32	0.08-65.07	.62	1.94	0.08-46.05	.68
Pre-treatment Tg (positive)	—	—	—	6.13	1.90-19.83	.002

## Discussion

In this retrospective study of 304 patients with DTC undergoing initial RAIA, we found that UICR was not associated with early disease outcomes. Although UICR and urinary iodine concentrations were significantly lower in patients prepared with THW compared with those receiving rhTSH, there was no difference in UICR between patients who were disease-free and those who experienced disease recurrence or persistence at 12 months. In multivariable analyses, younger age, advanced AJCC stage, and positive pre-treatment Tg were independent predictors of disease recurrence/persistence, whereas UICR and preparation method were not.

Early reports had suggested that high urinary iodine levels could impair radioactive iodine uptake, leading to incomplete remnant ablation or higher recurrence rates [11]. Sohn et al found that in DTC patients undergoing THW and a 2-week LID, ablation success was significantly affected only at extreme urinary iodine/creatinine ratios (UICR), specifically, when UICR was either <250 or >250 µg/gCr, suggesting iodine status impacts efficacy primarily at outlier levels [8]. More recent smaller studies with shorter follow up have challenged the predictive value of pre-ablation urinary iodine in adequately prepared patients [12, 13]. Our study, which included a larger cohort and a longer follow-up period than most previous reports, further supports these findings. Importantly, our patients were exceptionally well prepared, with uniformly low urinary iodine excretion following the LID. Even in the highest UICR quartile, the median UICR was 119.0 µg/gCr (IQR 90.7-176.2) which is well below levels previously associated with impaired ablation efficacy. Only 24 patients had a UICR greater than 150 µg/gCr, and among these, just 3 experienced disease recurrence or persistence. The small number of patients within this higher UICR range limited our ability to perform meaningful subgroup analyses. The narrow range of UICR likely reflects effective dietary counseling and strong adherence to the LID protocol. Because our follow-up period was longer, allowing for more complete identification of recurrence events, the absence of an association between UICR and disease recurrence/persistence provides strong evidence that once adequate iodine depletion is achieved before ablation, further variability in iodine excretion does not influence early post-ablation outcomes.

In our cohort, the median UICR values were low across both preparation groups, indicating that the majority of patients successfully achieved iodine depletion prior to ablation. The narrow range of UICR, particularly among THW-prepared patients, suggests consistent adherence to the LID protocol and limited dietary variability. This likely explains the absence of an association between UICR and recurrence/persistence. When all patients achieve sufficient iodine restriction, the UICR no longer discriminates between clinical outcomes. In other words, UICR may have limited prognostic value in well-prepared populations but may remain useful as a compliance indicator in settings where dietary control or iodine exposure is more variable.

The observed differences in UICR between THW and rhTSH preparation groups are biologically plausible. Patients undergoing THW appear to have similar renal clearance of iodine when hypothyroid after dietary depletion, whereas those receiving rhTSH preparation continue on LT4 and therefore have more stable iodine intake [14]. Continued use of LT4 contributes to this stable iodine intake, as iodine constitutes approximately 65% of the molecular weight of levothyroxine. Nevertheless, our data show that these biochemical differences did not translate into differences in disease outcomes, consistent with prior randomized trials showing equivalent efficacy of rhTSH and THW for remnant ablation and recurrence prevention when preparation is adequate [3].

Pre-treatment Tg emerged as a strong independent predictor of recurrence/persistence, consistent with existing literature demonstrating that elevated Tg before ablation reflects residual thyroid tissue or microscopic disease burden [15]. Likewise, advanced AJCC stage was associated with higher recurrence risk, highlighting the continued importance of tumor stage and baseline disease extent in outcome prediction [16]. The lack of an independent effect of the preparation method or UICR on disease outcomes suggests that iodine depletion beyond the recommended threshold may offer no incremental clinical benefit once minimal levels are achieved.

This is a relatively large sample size with a standardized institutional RAIA protocol, and comprehensive biochemical and staging data. The availability of both THW and rhTSH preparation groups also allowed for meaningful subgroup comparisons. However, several limitations warrant consideration. The overall UICR range was narrow, particularly among THW-prepared patients, reflecting effective but relatively uniform LID adherence. This homogeneity likely limited our ability to detect a potential relationship between iodine excretion and treatment outcomes. Follow-up was limited to 12 months, which may underestimate late recurrences or longer-term differences in outcomes. This was a single-center study from a region with moderate dietary iodine intake. Therefore, findings may not generalize to populations with higher baseline iodine exposure or variable dietary habits. Lastly, although pre-treatment Tg and TgAb were included in the analysis, we reported only qualitative results (positive/negative) rather than quantitative values due to the use of different assays over the study period. This approach was necessary to mitigate inter-assay variability but may have introduced some measurement bias. In addition, this binary classification of Tg may not fully capture the prognostic gradient associated with varying Tg levels.

In summary, UICR was not independently associated with differences in disease recurrence or persistence after initial RAIA in patients with DTC. In well-prepared patients achieving effective iodine depletion, UICR demonstrated limited variability and did not predict early disease outcomes. UICR measurement in well prepared patients may serve best as a compliance marker for the LID rather than as a prognostic biomarker for recurrence or ablation success. Future studies should explore whether UICR variability predicts outcomes in populations with higher iodine intake, less dietary control, or variable compliance.

## Data Availability

Deidentified data supporting the findings of this study are available upon reasonable request to the corresponding author.

## Abbreviations

AJCC: American Joint Committee on Cancer
BIDMC: Beth Israel Deaconess Medical Center
DTC: differentiated thyroid cancer
LID: low-iodine diet
NIS: sodium–iodide symporters
OR: odds ratio
RAIA: radioactive iodine ablation
rhTSH: recombinant human TSH
THW: thyroid hormone withdrawal
TSH: thyroid-stimulating hormone
UICR: urinary iodine-to-creatinine ratio
